# Major Vessels of the Interclavicular Suprasternal Space: A Suprasternal Notch-Referenced Topographic Classification with Potential Procedural Relevance

**DOI:** 10.3390/diagnostics16172724

**Published:** 2026-08-26

**Authors:** Ionuț Mădălin Munteanu, Adelina Maria Jianu, Răzvan Costin Tudose, Mugurel Constantin Rusu, Alexandra Diana Vrapciu, George Triantafyllou, Maria Piagkou, Iulian Brezean

**Affiliations:** 1 Clinic of Anaesthesia and Intensive Care, Institute for Cardiovascular Diseases of Timișoara, “Victor Babeș” University of Medicine and Pharmacy, 300310 Timișoara, Romania; madaimunteanu@gmail.com; 2 Faculty of Medicine, “Victor Babeș” University of Medicine and Pharmacy, 300041 Timișoara, Romania; adelina.jianu@umft.ro; 3 Department of Radiology and Medical Imaging, Emergency Clinical Municipal Hospital of Timișoara, 300254 Timișoara, Romania; 4 Division of Anatomy, Department 1, Faculty of Dentistry, “Carol Davila” University of Medicine and Pharmacy, 050474 Bucharest, Romania; razvan-costin.tudose0721@stud.umfcd.ro (R.C.T.); alexandra.vrapciu@umfcd.ro (A.D.V.); 5 Department of Anatomy, School of Medicine, Faculty of Health Sciences, National and Kapodistrian University of Athens, 75 Mikras Asias Str., Goudi, 11527 Athens, Greece; georgerose406@gmail.com (G.T.); piagkoumara@gmail.com (M.P.); 6 Discipline of General Surgery and Qualified Care in Surgical Specialties, Department of Specific Disciplines, Faculty of Midwifery and Nursing, “Carol Davila” University of Medicine and Pharmacy, 050474 Bucharest, Romania; iulian.brezean@umfcd.ro; 7 Department of General Surgery, “Dr. Ion Cantacuzino” Clinical Hospital, 030167 Bucharest, Romania

**Keywords:** aortic arch, brachiocephalic trunk, left brachiocephalic vein, suprasternal notch, sternoclavicular joint, computed tomography angiography, high-riding left brachiocephalic vein

## Abstract

**Background/Objectives**: The suprasternal notch (SN) is the conventional landmark for front-of-neck airway access and anterior cervical surgery, yet the great vessels may extend to or above it. To our knowledge, no previous study has jointly classified the aortic arch (AA), brachiocephalic trunk (BCT), left brachiocephalic vein (LBCV), and sternoclavicular joint (SCJ) configuration relative to the SN in a single adult cohort. **Methods**: In this single-centre retrospective study, 100 consecutive adult computed tomography angiography (CTA) studies were reviewed. Using a horizontal SN reference plane, the AA, BCT, and LBCV were each graded as mediastinal, cervicomediastinal, or cervical/high-riding; the bilateral SCJ manubrial joining grade was scored on four levels and integrated into three categories (mild, clear, marked). Two observers independently classified all cases (weighted Cohen kappa = 0.89). Associations were assessed with Spearman correlation and chi-square tests. **Results**: The BCT was the most variable structure, cervical/high-riding in 49% and reaching or crossing the SN in 64%; the AA and LBCV were cervical/high-riding in 3% and 5%, respectively. Complete SCJ manubrial joining was absent in all cases, and the integrated SN space was marked in 15% of cases. Cervical/high-riding BCT was more frequent in women (63.5% vs. 33.3%; *p* = 0.005). Five of six pairwise correlations remained significant after Benjamini–Hochberg adjustment, with the strongest association for AA–BCT (rho = 0.406). **Conclusions**: The BCT was the principal great vessel extending to or above the SN in this cohort, whereas cervical/high-riding AA and LBCV were uncommon. The SN-referenced classification provides a reproducible descriptive framework for this topography. These findings represent cohort frequencies rather than population-based prevalence estimates, and their potential procedural relevance requires prospective validation.

## 1. Introduction

The suprasternal notch (SN)—termed the jugular notch of the sternum in the current Terminologia Anatomica, with suprasternal notch retained as an accepted synonym—is conventionally defined as the uppermost midline concavity of the manubrium sterni, but its actual extent as an interclavicular space is modified by the sternal ends of the clavicles and their degree of articulation with the manubrium. In chest computed tomography (CT) studies of asymptomatic adults, the sternoclavicular joints (SCJ) show substantial normal variability and asymmetry; Tuscano et al. (2009) specifically defined poor or limited articulation as less than 25% of the clavicular cortical surface articulating with the manubrium and found this configuration in 5 of 104 patients [1]. Therefore, in some individuals, the SN is better regarded as a suprasternal–interclavicular space rather than a narrow midline notch [2,3].

The BCT is normally the first branch of the aortic arch, arising within the superior mediastinum and ascending obliquely to the right to bifurcate behind the right sternoclavicular joint, so that in most adults it lies below the SN. This space is clinically relevant because major vessels may lie anterior to the trachea at, or just above, the SN. In 500 adult thoracic CT scans, Weightman and Gibbs (2018) found a major vessel anterior to the trachea in 53% of cases at the SN, in 25% at 10 mm above it, in 9% at 20 mm above it and in 1% at 30 mm above it, most often involving the brachiocephalic trunk (BCT) [4]. The case-based surgical literature reinforces this risk of encountering the BCT anterior to the cervical trachea: Zarokosta et al. (2019) described an aberrantly high BCT encountered during parathyroid adenoma excision, with bifurcation anterior to the cervical trachea [5]. Such configurations are relevant to emergency front-of-neck access, tracheostomy and thyroid or parathyroid surgery.

The aortic arch (AA) and its branches also show substantial morphological variability. In a consecutive adult computed tomography angiography (CTA) series, Berko et al. (2009) reported normal arch branching in 66.0%, arch branching variants in 32.4% and anomalous branching in 1.5%; common BCT-left common carotid origin accounted for 27.4%, direct left vertebral artery origin for 6.6% and aberrant right subclavian artery for 1.2% [6]. Review articles based on multidetector CT similarly emphasise that cross-sectional imaging is suited to depicting arch-sidedness, branching patterns and spatial relationships with the trachea and oesophagus [7,8]. A cervical aortic arch is a distinct congenital entity in which the arch is located at or above the clavicular level and may protrude into the neck, but this rare diagnosis should be distinguished from simpler SN-referenced elevation of the arch apex [7,9].

The left brachiocephalic vein (LBCV) is another structure that may approach the suprasternal region. In its usual configuration, it forms from the left internal jugular and subclavian veins, crosses the upper mediastinum anterior to the aortic arch branches and dorsal to the thymus, and joins the right brachiocephalic vein to form the superior vena cava [10]. Compared with arterial branching variants, central venous anomalies are uncommon in adult CTA series: Berko et al. (2009) found central venous anomalies in 0.7% of 1000 adult CT angiograms, including one retroaortic brachiocephalic vein [6]. However, the ordinary vertical relationship of the LBCV to the SN has not been systematically classified in adult CTA cohorts.

Clinically, front-of-neck airway access, tracheostomy, and anterior cervical, thyroid and parathyroid surgery are all planned on the assumption that the great vessels lie safely below the SN. Whether this assumption holds, and whether arterial, venous and skeletal deviations from it tend to cluster in the same patients, has not been tested jointly. Quantifying how often, and in which combinations, these structures reach the interclavicular space is therefore directly relevant to pre-procedural anatomical assessment.

To our knowledge, no previous adult CTA study has jointly classified the positions of the AA, BCT, LBCV, and SCJ manubrial-joining grade relative to the SN in a single cohort and statistically analysed their co-distribution. The present study addresses this gap by applying a standardised ordinal topographic classification to these four variables on CTA, describing their individual and combined frequency distributions, and identifying inter-variable associations.

## 2. Materials and Methods

### 2.1. Study Design and Patient Population

A retrospective observational study was conducted. One hundred consecutive adults were screened sequentially from the institutional CTA archive between February and June 2026. The specific original clinical indications were not consistently recoverable from the archived imaging dataset and therefore could not be characterised retrospectively. Pre-specified exclusion criteria (inadequate image quality, prior thoracic surgery, or incomplete visualisation of the suprasternal region) were applied, but no screened case met these criteria; accordingly, all 100 consecutively screened patients were included and none were excluded. The study was conducted in accordance with institutional ethical standards. The study was approved by the Ethics Committee of the Emergency Clinical Municipal Hospital of Timișoara (approval number E-4247, 15 October 2025). Informed consent was obtained from all subjects involved in the study. Patient identifiers were retained only in the internal classification database and are not disclosed in the published report.

Patient sex and age at the time of CTA were recorded. All cases were independently classified by two trained observers (M.C.R. and A.M.J.), each blinded to the other’s assignments and to clinical data. Discrepancies were resolved by consensus, and the consensus classification was used for all subsequent analyses. Findings were entered into a structured classification template developed specifically for this study. The cohort comprised 100 consecutive adults—48 men (48%) and 52 women (52%); mean age at CTA was 66.5 ± 12.8 years (range 36–90). Age did not differ significantly between the sexes (men 66.2 ± 12.5 years; women 66.7 ± 13.3 years; independent-samples *t* test, t = 0.19, *p* = 0.85).

### 2.2. CT Angiography

All examinations were performed on multidetector CT scanners used in routine clinical care. Because the study retrospectively analysed archived CTA datasets acquired using heterogeneous clinical protocols, the scanner manufacturer/model and the specific commercial iodinated contrast-agent product were not consistently recoverable from the study archive and therefore could not be reported reliably. Intravenous iodinated contrast medium was administered via an antecubital vein at a standard flow rate, with image acquisition timed to the arterial or venous phase as dictated by the clinical indication. Axial images were acquired with a nominal slice thickness of ≤1 mm and reconstructed in coronal and oblique sagittal planes with multiplanar reformation (MPR). No dedicated imaging protocol was established for this study; images were reviewed as originally acquired. Because the primary endpoint was categorical craniocaudal position relative to a fixed bony landmark (the SN), rather than vessel attenuation or a millimetric vascular measurement, protocol heterogeneity was expected to have less influence on classification than on quantitative vascular analysis. Nevertheless, differences in scanner platform and arterial-versus-venous contrast timing may have affected vessel conspicuity, particularly for the LBCV; a dedicated protocol would be required for metric analyses such as vessel-to-trachea distance. The use of thin-slice multiplanar CTA is consistent with contemporary CT-based evaluation of the adult aorta and aortic arch variants, where multiplanar and three-dimensional reconstructions are used to define vessel course and relationships to the trachea, oesophagus and thoracic inlet [7,8,11].

### 2.3. Image Analysis

DICOM datasets were reviewed using Horos, version 3.3.6 (an open-source DICOM viewer; Horos Project, https://www.horosproject.org/). For each case, axial, coronal and oblique sagittal reconstructions were reviewed. The coronal plane served as the primary reference for determining the craniocaudal relationship of each vascular structure to the suprasternal notch (SN). Final entries were categorical. No continuous metric measurements were retained for analysis; SCJ grade was assigned by categorical estimation of the manubrial joining proportion on coronal images. Each observer reviewed and classified all cases independently in separate sessions, without access to the other observer’s entries.

### 2.4. Anatomical Reference Level

The SN was defined as the uppermost margin of the manubrium sterni at the midline, identifiable on both coronal and sagittal reformations as the apex of the manubrial concavity. This point served as the fixed topographic reference for classifying all vascular structures and the sternoclavicular joint (SCJ) configuration. A horizontal plane through this point, the SN reference plane, was used to determine whether a given vascular structure lay below, crossed, or extended above this level. This approach is conceptually related to CT-based SN-level assessment of pretracheal major vessels [4], but the present study retained ordinal categorical entries rather than continuous millimetric distances.

### 2.5. Topographic Classification System

Four anatomical variables were classified for each case: the AA, BCT, LBCV, and bilateral SCJ manubrial joining grade. Each vascular structure received an ordinal code on a three-level scale (codes 1–3); the SCJ was graded on a four-level scale (codes 1–4). The AA × BCT combination was retained in the main analysis, while the global AA × BCT × LBCV × SCJ enumeration was retained descriptively in the Supplementary Materials.

AA classification. The AA was classified relative to the SN in three categories. Code 1, mediastinal: the AA lies entirely below the SN reference plane. Code 2, cervicomediastinal: the AA crosses the SN reference plane, lying partly above and partly below it. Code 3, cervical/high-riding: the AA lies entirely above the SN reference plane.

BCT and LBCV. Both structures were classified independently using the same three-level ordinal scale. Code 1, mediastinal: the vessel is entirely below the SN. Code 2, cervicomediastinal: the vessel crosses the SN reference plane, lying partly above and partly below it. Code 3, cervical/high-riding: the vessel lies entirely above the SN plane.

SCJ manubrial joining grade. The proportion of the sternal end of the clavicle in contact with the manubrium was graded bilaterally on coronal reconstructions. Code 1: complete (100%) manubrial joining, corresponding to classical suprasternal notch topography. Code 2: ≥50% to <100% of the sternal end of the clavicle joined to the manubrium, corresponding to a mild suprasternal–interclavicular component. Code 3: ≥25% to <50% joining, corresponding to a clear suprasternal–interclavicular component. Code 4: <25% joining, corresponding to a marked suprasternal–interclavicular space. No standardised grading system exists for the degree of sternoclavicular articulation; the four-level scale applied here is operational and was defined a priori for this study, anchored to the <25% cortical-contact threshold used by Tuscano et al. to denote poor articulation. The topographic interpretation of the SN region was derived automatically from the more severe of the two sides (right or left). For combination analyses, this integrated SCJ interpretation was represented as a three-level variable: mild, clear and marked.

Combined classification types. The combined scheme was designed as an analytical and descriptive layer applied to the individual SN-referenced grades, not as a predictive model or a new morphological taxonomy. For the AA × BCT axis, the nine theoretically possible combinations were assigned fixed codebook letters: A, mediastinal AA with mediastinal BCT; B, mediastinal AA with cervicomediastinal BCT; C, mediastinal AA with cervical/high-riding BCT; D, cervicomediastinal AA with mediastinal BCT; E, cervicomediastinal AA with cervicomediastinal BCT; F, cervicomediastinal AA with cervical/high-riding BCT; G, cervical/high-riding AA with mediastinal BCT; H, cervical/high-riding AA with cervicomediastinal BCT; and I, cervical/high-riding AA with cervical/high-riding BCT. The global four-variable combinations were enumerated only descriptively in Table S1 and Figure S2; no inferential testing was applied to individual four-variable types. For this supplementary enumeration, tier I comprised patterns with all vascular variables’ mediastinal and mild SCJ topography; tier II comprised patterns with cervicomediastinal vascular or clear SCJ involvement but no cervical/high-riding vessel and no marked SCJ; and tier III comprised patterns with at least one cervical/high-riding vessel or marked SCJ. Lowercase letters enumerate distinct patterns within each tier and imply no severity ranking among themselves.

### 2.6. Statistical Analysis

All analyses were performed in Python 3.13.5 using SciPy version 1.17.0 and scikit-learn version 1.8.0 for weighted Cohen kappa calculations. Continuous variables are reported as mean ± standard deviation (SD) with range; categorical variables are reported as absolute frequencies and percentages. The full three-category BCT distribution by sex was assessed with the chi-square test, and the specific 2 × 2 comparison of cervical/high-riding BCT by sex used the Yates-corrected chi-square. Spearman’s rank correlation coefficient (rho) was computed for the six pairs of ordinal anatomical variables. A two-tailed significance threshold of alpha = 0.05 was applied, and the six pairwise correlation *p*-values were adjusted using the Benjamini–Hochberg false-discovery-rate procedure. No inferential testing was applied to individual four-variable phenotypes. Inter-observer agreement for the ordinal topographic classifications was quantified using the weighted Cohen kappa coefficient (κw, linear weights). Kappa was interpreted according to Landis and Koch [12], with values above 0.81 denoting almost perfect agreement.

### 2.7. Comparative Literature Synthesis (Tables S2 and S3)

The comparative data on classical cervical aortic arch (Table S2) and bovine aortic arch (Table S3) were assembled as a structured narrative synthesis rather than de novo measurement, and were prepared in accordance with the quality criteria of the Scale for the Assessment of Narrative Review Articles (SANRA) [13], particularly its items on the description of the literature search and referencing. PubMed and Google Scholar, supplemented by the Consensus literature-search engine (which indexes PubMed and Semantic Scholar), were searched for English-language, peer-reviewed studies on the definition, classification, epidemiology, and clinical outcomes of cervical and bovine aortic arch, using combinations of the terms “cervical aortic arch,” “bovine aortic arch,” “aortic arch branching variant,” “prevalence,” “classification,” “meta-analysis,” and “outcome.” Only PubMed-indexed full-text articles were retained; conference abstracts, preprints, and non-indexed or predatory-journal sources were excluded. When several sources addressed the same parameter, the largest series or the most recent meta-analysis or individual-patient-data review was preferred. Reported values were extracted directly from the selected studies and are cited in the corresponding table rows; no pooled re-analysis of individual patient data was undertaken, and no formal risk-of-bias assessment or PRISMA systematic-review protocol was applied, consistent with the narrative nature of these tables.

## 3. Results

### 3.1. Inter-Observer Reliability

Inter-observer agreement was almost perfect for all four classifications (weighted kappa: AA = 0.93, BCT = 0.91, LBCV = 0.87, SCJ = 0.85; mean 0.89).

### 3.2. Aortic Arch Position Relative to the Suprasternal Notch

Using the SN-referenced classification described above, the AA was mediastinal in 85 cases (85%), cervicomediastinal in 12 cases (12%) and cervical/high-riding in 3 cases (3%) (Table 1). The AA never occupied a topographically higher category than the BCT in this cohort.

### 3.3. Brachiocephalic Trunk Position Relative to the Suprasternal Notch

The BCT was mediastinal in 36 cases (36%), cervicomediastinal in 15 (15%) and cervical/high-riding (HR BCT) in 49 (49%) (Table 2). The full three-category BCT distribution differed by sex (chi-square = 11.267, df [degrees of freedom] = 2, *p* = 0.004). In the binary comparison, HR BCT was more frequent in women than in men: 33 of 52 women (63.5%) versus 16 of 48 men (33.3%; Yates-corrected chi-square = 7.901, *p* = 0.005). In 61 of 100 cases (61%), the BCT lay in a higher SN-referenced category than the AA (combination types B, C and F in Table 3); the AA never exceeded the BCT, and the two structures shared the same category in the remaining 39 cases (types A and I).

### 3.4. AA × BCT Combined Classification

Five of nine theoretically possible AA × BCT combination types were observed (Table 3). Spreadsheet codebook combinations D, G and H, all of which would place the BCT in a lower topographic category than the AA, and combination E (cervicomediastinal AA with cervicomediastinal BCT), were absent; all nine AA × BCT codes are defined in the Topographic Classification System (Section 2.5). AA and BCT categories were positively associated (Spearman rho = 0.406, *p* < 0.001). Types A/Ia and C/IIIa together accounted for 70% of the cohort.

**Figure 1:**
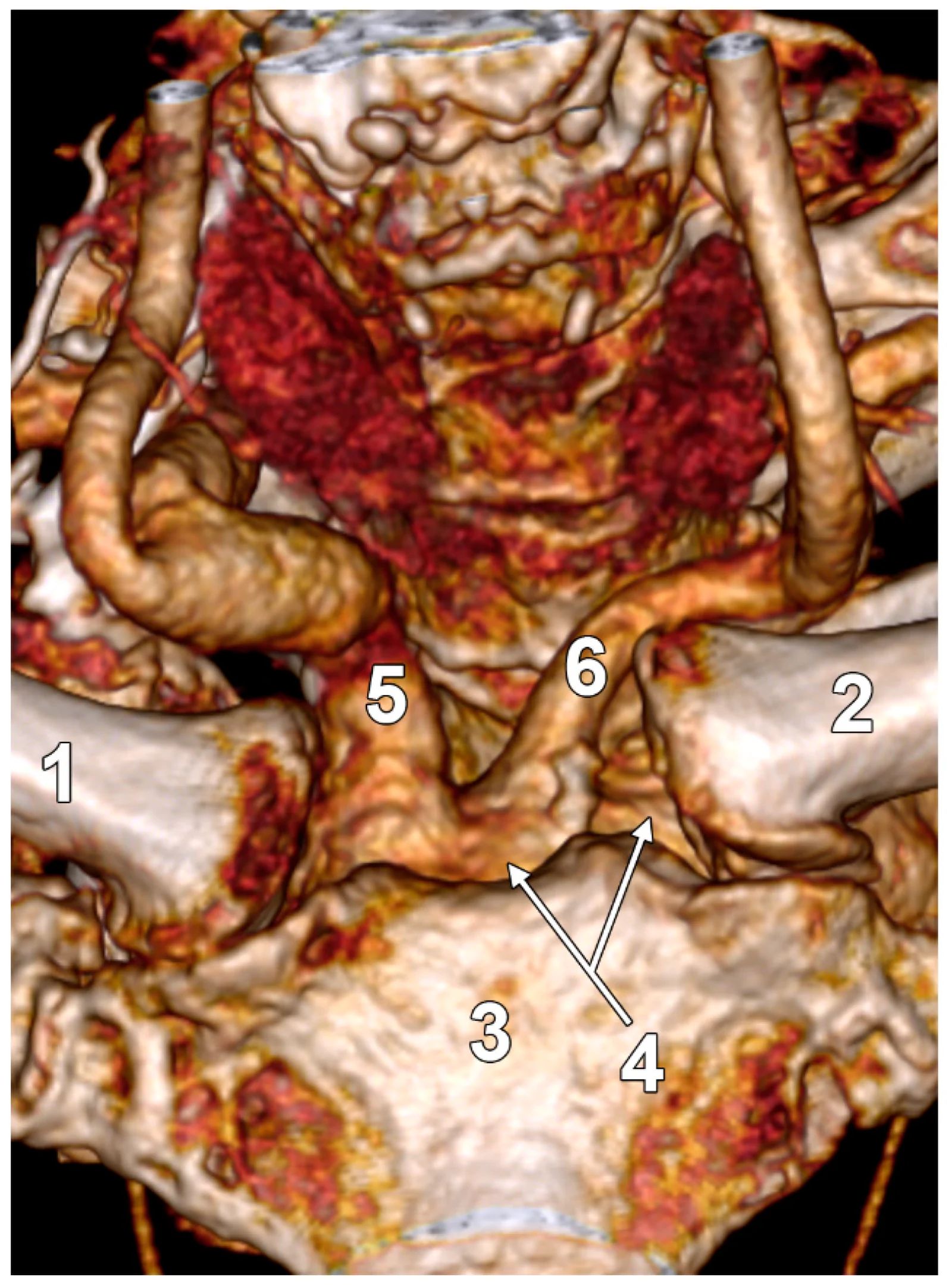
Three-dimensional volume-rendered CTA reconstruction showing a combination F topographic pattern: cervicomediastinal aortic arch (AA code 2, apex crossing the suprasternal notch plane) with a cervical/high-riding brachiocephalic trunk (BCT code 3, entirely above the suprasternal notch reference plane). Anterior view. The interclavicular suprasternal space simultaneously contains the upper margin of the AA and the full cervical course of the BCT anterior to the trachea. 1. Right clavicle; 2. left clavicle; 3. sternal manubrium; 4. aortic arch; 5. brachiocephalic trunk; 6. left common carotid artery.

**Figure 2:**
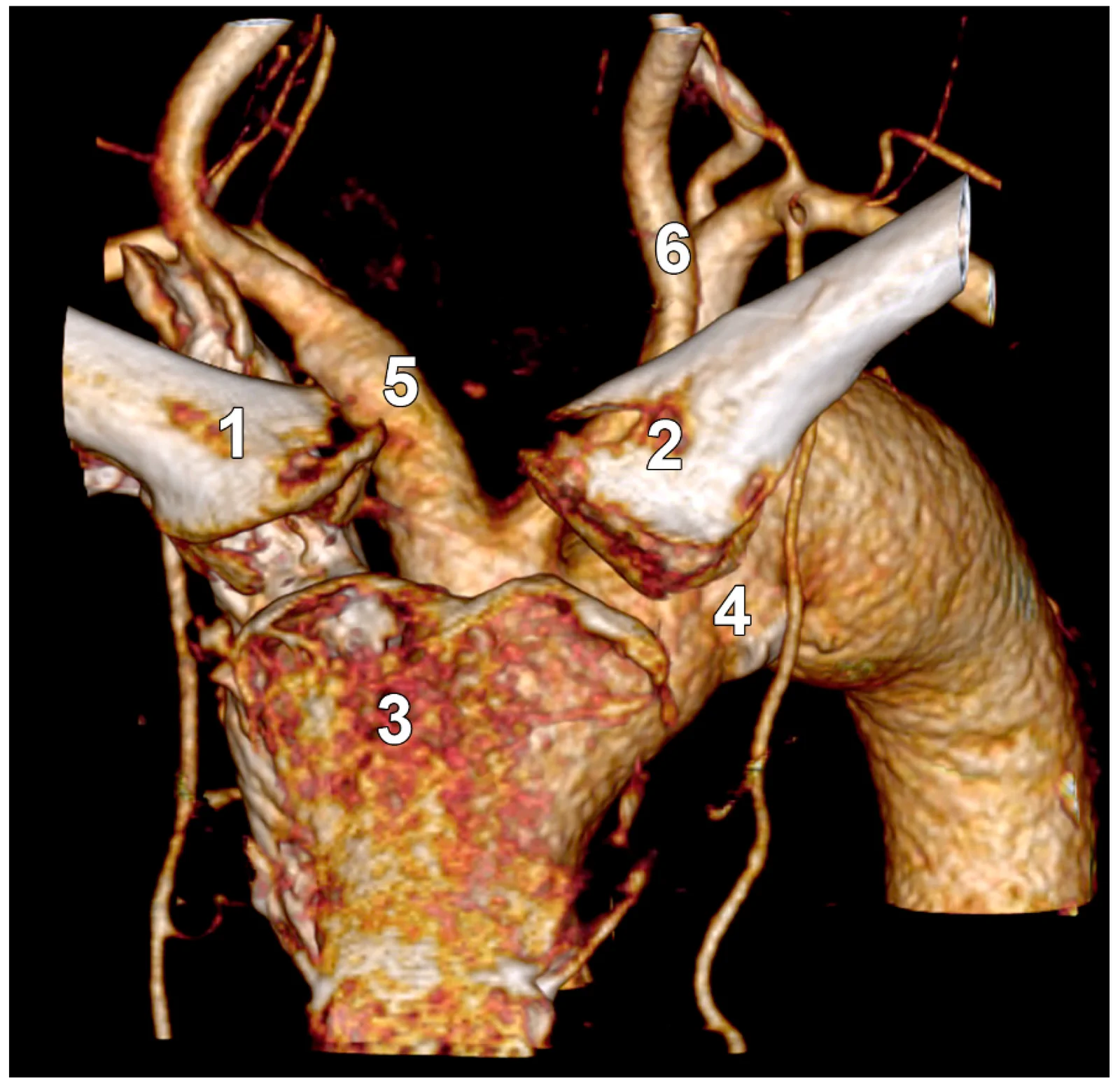
Three-dimensional volume-rendered CTA reconstruction showing a combination I topographic pattern (type IIIc): cervical/high-riding aortic arch (AA code 3, lying entirely above the suprasternal notch reference plane) with a cervical/high-riding brachiocephalic trunk (BCT code 3, entirely above the suprasternal notch reference plane). Common innominate—left common carotid origin trunk (bovine aortic arch). Anterior view. It represents the most elevated AA × BCT topographic phenotype in this series. 1. Right clavicle; 2. left clavicle; 3. sternal manubrium; 4. aortic arch; 5. brachiocephalic trunk; 6. left common carotid artery.

The combination F pattern (cervicomediastinal AA with cervical/high-riding BCT, type IIIb; 12 cases, 12%) is illustrated in Figure 1. It is the second most common Tier III pattern in the cohort, after combination C (mediastinal AA + cervical/high-riding BCT, 34 cases). The most extreme combination, type IIIc, cervical/high-riding AA with cervical/high-riding BCT, was identified in 3 cases (3%) and is illustrated by three-dimensional volume-rendered CTA in Figure 2.

### 3.5. Left Brachiocephalic Vein Position Relative to the Suprasternal Notch

The LBCV was mediastinal in 73 cases (73%), cervicomediastinal in 22 (22%) and cervical/high-riding in 5 (5%) (Table 4). All five cervical/high-riding LBCV cases occurred within the HR BCT subgroup; in this cohort, LBCV cervical elevation was not observed independently of BCT elevation. A significant positive association between LBCV and BCT categories was observed (chi-square = 16.261, *p* = 0.003; Spearman rho = 0.388, *p* < 0.001, df = 4).

### 3.6. Sternoclavicular Joint Topography and Suprasternal Notch Configuration

No case in this series showed complete (100%) manubrial joining of either SCJ, showing that classical suprasternal notch morphology, as defined here, was absent in this cohort. Right and left SCJ grades are shown in Table 5. Bilateral symmetry was high: right and left grades were identical in 94 of 100 cases (94%). The integrated topographic interpretation, derived from the more severe side, is shown in Table 6. Among the 15 cases with a marked suprasternal–interclavicular space, 11 (73.3%) had HR BCT. A significant positive correlation was found between BCT category and SCJ grade (Spearman’s rho = 0.282, *p* = 0.004). The LBCV-SCJ pair showed no significant association (rho = 0.152, *p* = 0.132).

### 3.7. Pairwise Correlations and Descriptive Four-Variable Enumeration

**Figure 3:**
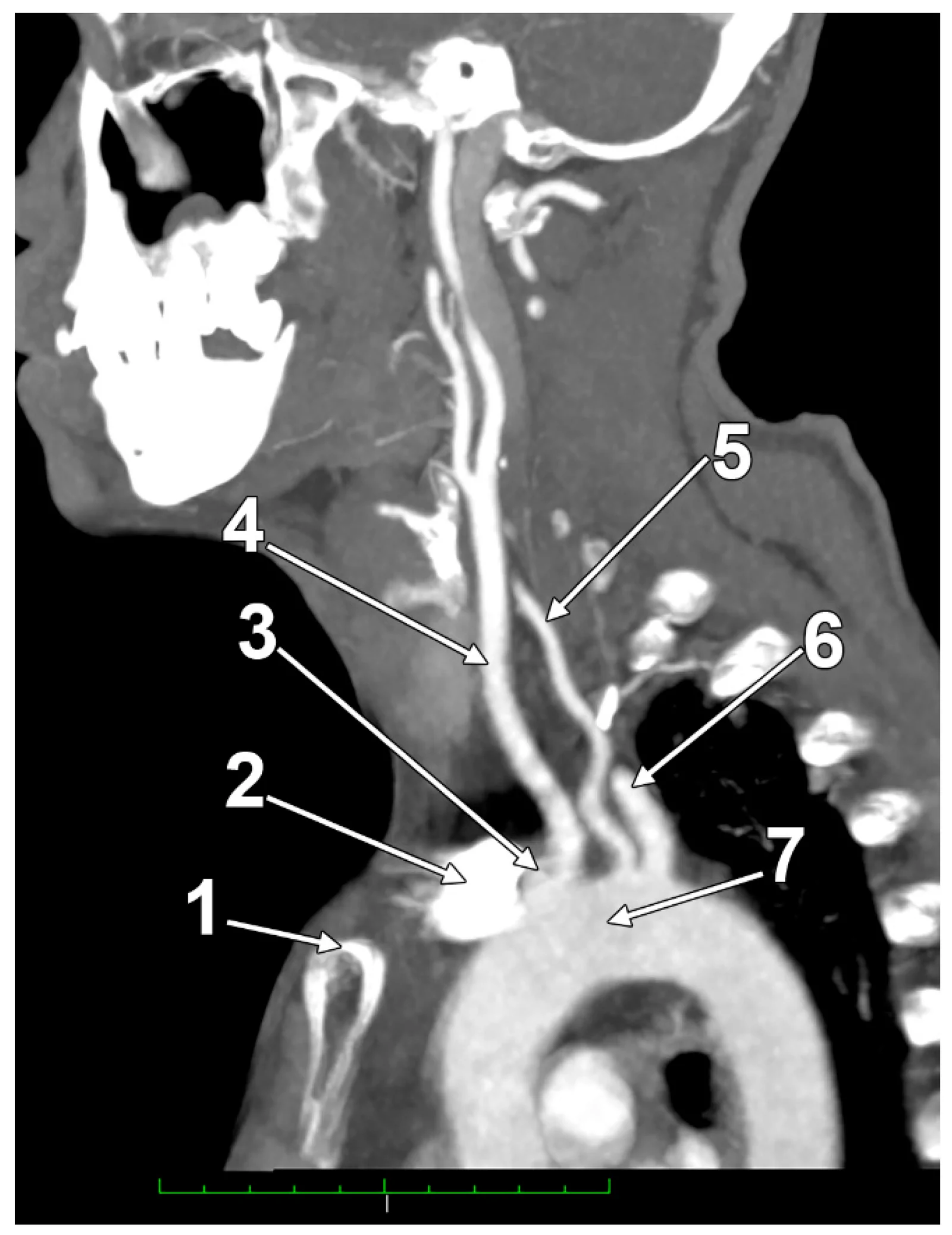
Global type IIIr, with the AA, BCT and LBCV all simultaneously cervical/high-riding. Common innominate—left common carotid origin trunk (bovine aortic arch). Aortic origin of the left vertebral artery. Midsagittal slice through neck and upper mediastinum. Left lateral view. 1. Suprasternal notch; 2. left brachiocephalic vein; 3. brachiocephalic trunk; 4. left common carotid artery; 5. left vertebral artery; 6. left subclavian artery; 7. aortic arch.

All six pairwise ordinal correlations were examined. Five were significant before and after Benjamini–Hochberg adjustment, with AA–BCT yielding the strongest association (rho = 0.406, *p* < 0.001); the LBCV–SCJ pair was not significant (rho = 0.152, *p* = 0.132). The global four-variable enumeration yielded 26 descriptive topographic types (Table S1; Figure S2). Because many were represented by a single case, no inferential testing was applied to individual four-variable types. The most frequent global type was Ia (n = 17), whereas type IIIr, with AA, BCT and LBCV all being cervical/high-riding and a clear SCJ space, was observed once (Figure 3).

## 4. Discussion

### 4.1. Principal Findings

**Figure 4:**
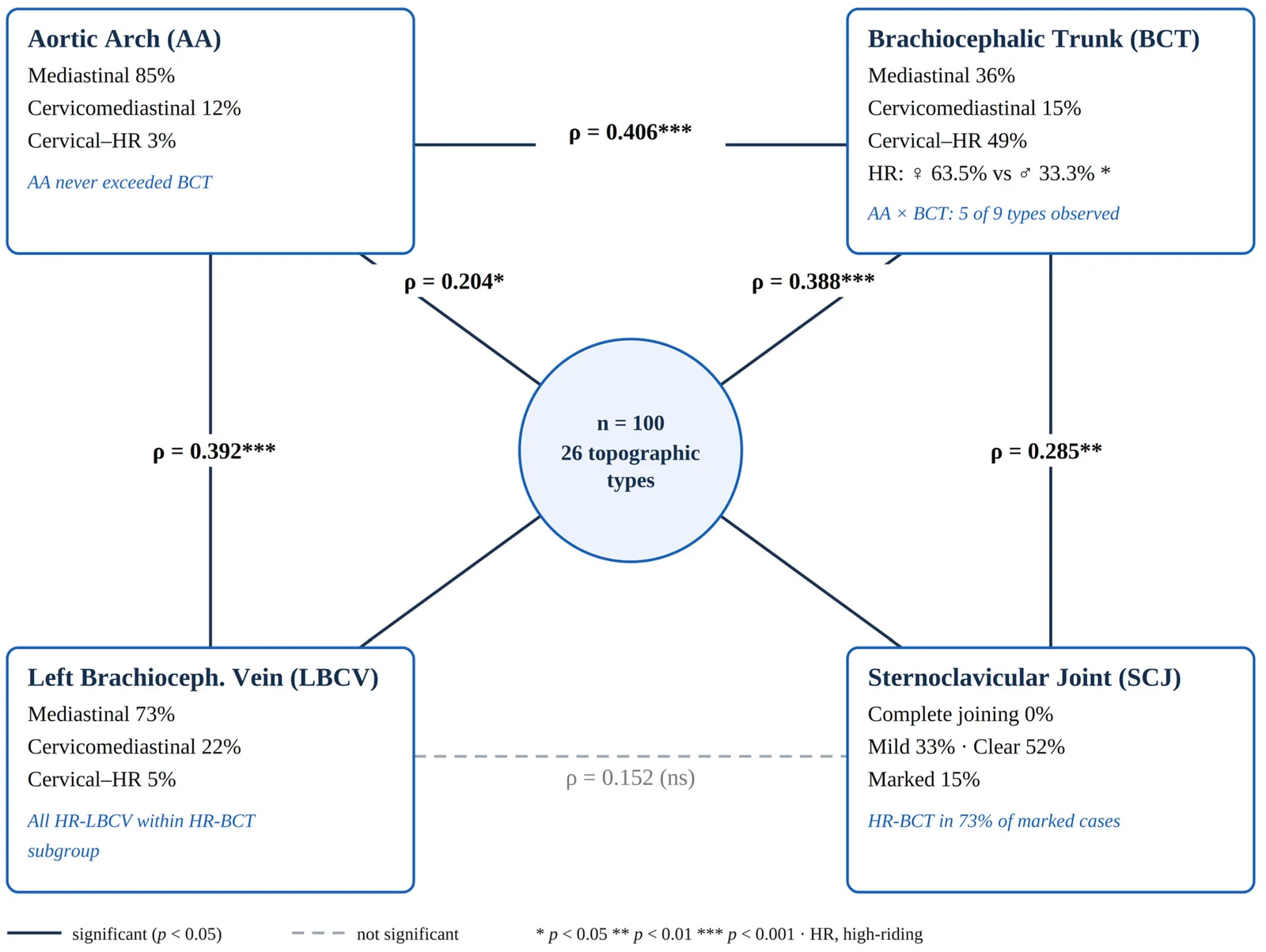
Summary diagram of the suprasternal–interclavicular topographic system and inter-variable associations. Diagram summarising the four analysed variables in 100 CTA cases: aortic arch (AA), brachiocephalic trunk (BCT), left brachiocephalic vein (LBCV), and integrated sternoclavicular joint (SCJ) topography. Each box reports the category distribution relative to the suprasternal notch reference plane or, for SCJ, the integrated bilateral manubrial joining grade. Connecting lines show Spearman’s rank correlations between ordinal variables. Five of the six pairwise correlations were significant. The strongest positive association was observed between AA and BCT position (ρ = 0.406, *p* < 0.001), followed by AA-LBCV (ρ = 0.392, *p* < 0.001), BCT-LBCV (ρ = 0.388, *p* < 0.001), BCT-SCJ (ρ = 0.282, *p* = 0.004), and AA-SCJ (ρ = 0.204, *p* = 0.04). The LBCV-SCJ association was not significant (ρ = 0.152). HR, high-riding; ns, not significant. Raw *p*-values are displayed; the significance pattern was unchanged after Benjamini–Hochberg adjustment of the six pairwise correlations.

This study applies an SN-referenced topographic classification of the AA, BCT, LBCV and SCJ manubrial joining grade in 100 adult CTA studies (Figure 4). The BCT was the most variable structure, with 49% of cases classified as cervical/high-riding and 64% showing at least some extension to or above the SN plane when cervicomediastinal and cervical/high-riding categories were pooled. The AA was mediastinal in 85%, cervicomediastinal in 12% and cervical/high-riding in 3%, while the LBCV was mediastinal in 73%, cervicomediastinal in 22% and cervical/high-riding in 5%. Complete (100%) manubrial SCJ joining was absent; the integrated SN topographic space was mild in 33%, clear in 52% and marked in 15%. Five of the six pairwise Spearman correlations were significant, the exception being the LBCV-SCJ pair; this significance pattern was unchanged after Benjamini–Hochberg adjustment. The results extend prior CT-based work on pretracheal major vessels by adding a parallel categorical assessment of the AA, BCT, LBCV, and SCJ configurations [4].

### 4.2. AA Position and Arch Variant Context

The AA was predominantly mediastinal (85%), and the three cervical/high-riding cases should not be equated with classical cervical aortic arch (CAA), a congenital anomaly defined by an arch at or above the clavicular level [7,9]. MDCT can distinguish arch morphology and its relationships to adjacent structures [8]. Accordingly, the present SN category is purely topographic and complements, rather than replaces, conventional arch classification and CTA reporting frameworks [7,11].

Classical CAA is rare; representative adult surgical data are summarised in Table S2 [14]. Adult case-review data are also included [15]. Reported clinical presentations [16,17] and the Haughton classification [18] are retained there for context. The three cervical/high-riding AA cases in the present series exceeded the SN plane but did not reach the clavicular level and therefore represent a distinct SN-referenced topographic finding.

The so-called bovine aortic arch is a common adult branching variant [19]. Its prevalence has been quantified in large meta-analyses [20], while its terminology [21] and broader associations with thoracic aortic disease [22] and TAVR outcomes [23] have been reported elsewhere. Because bovine arch morphology was not classified in the present cohort, these data are retained only as contextual material in Table S3.

### 4.3. BCT Position: Threshold Dependence and Cohort Frequency

Cervical/high-riding BCT occurred in 49% of this cohort, and 64% reached or crossed the SN plane. These are topographic cohort frequencies rather than population prevalence estimates. Broader CTA data distinguish common arch variants from rarer anomalies [6]; the closest external comparator found a major pretracheal vessel at the SN in 53% of adults [4]. Isolated surgical reports document high BCT encountered during cervical procedures [5], but such reports provide clinical context rather than outcome validation for the present classification.

Additional reports describe high BCT relationships to the upper tracheal rings [24], intraoperative modification of the approach [25], compressive symptoms, including dysphagia [26] or respiratory compromise [27], and associated vascular variants [28,29]. These observations are contextual and were not outcomes assessed in this cohort.

Doppler ultrasonography can depict suspected high BCT anatomy [30]; its role relative to the present CTA classification requires prospective evaluation.

The suprasternal view can also visualise the aortic arch and branch origins [31]. Whether a higher SN-referenced grade predicts sonographic accessibility or any procedural outcome remains to be tested prospectively.

### 4.4. Interclavicular Suprasternal Space and Topographic Relevance

Normal SCJ variability and the <25% articulation threshold are documented in the CT literature [1]. In this cohort, complete joining was absent, 15% had a marked suprasternal–interclavicular space, and 73.3% of those marked cases had a cervical/high-riding BCT. A marked space may therefore serve as an anatomical cue for deliberate review of the BCT and LBCV on CTA, but no procedural outcomes were assessed and prospective validation is required. This interpretation is anatomically consistent with prior pretracheal-vessel and surgical observations [4,5].

### 4.5. Sex Differences

The BCT category distribution differed by sex, and cervical/high-riding BCT was more frequent in women than in men (63.5% versus 33.3%; Yates-corrected chi-square = 7.901, *p* = 0.005). This cohort-level association should not be interpreted as causal. Differences in thoracic inlet geometry may contribute, but these were not measured in the present study and require validation in larger sex-stratified CTA series.

### 4.6. LBCV and SCJ Independence

BCT and LBCV positions were positively correlated (rho = 0.388, *p* < 0.001), and all five cervical/high-riding LBCV cases occurred within the HR BCT subgroup. This SN-referenced vertical category should not be conflated with congenital LBCV anomalies described in the prenatal literature [10] or with the rare central venous anomalies reported in adult CTA series [6]. The LBCV-SCJ association was not significant (rho = 0.152, *p* = 0.132).

No established study defines “high-riding LBCV” as a clinical entity. A case of BCT-related LBCV compression provides limited contextual support [32], but no haemo-dynamic, surgical, or procedural outcomes were assessed in the present cohort.

The other literature describes the relevance of variant brachiocephalic venous anatomy to central venous catheterisation [33], difficult catheter or port placement [33,34], tracheostomy-related venous injury [35], and altered surgical planning [36]; these reports are contextual only and were not study outcomes.

### 4.7. Combination Classification System

The tiered-and-lettered combination classification provides a concise notation for multi-variable SN topographic profiles. In the main manuscript, the AA × BCT combination is retained because it directly captures the anatomical relationship between the arch and its first branch. The full four-variable enumeration is presented only descriptively in Table S1 and Figure S2 and is not intended as a predictive model. The single type IIIr case represents the most anatomically crowded topographic configuration observed, with the AA, BCT and LBCV all cervical/high-riding in the pretracheal interclavicular plane.

### 4.8. Limitations

This study has several limitations. The observed percentages only describe this single-centre retrospective sample and must not be interpreted as population-based prevalence estimates. The heterogeneous CTA protocols and non-randomised sampling frame further limit generalisability. The SN-referenced definitions for AA, BCT and LBCV position, as well as the integrated SCJ category, are operational and have not been externally validated. The study used ordinal categories rather than continuous vertical measurements and did not classify the broader spectrum of arch and branch variants described in other frameworks [4,6,7,11]. Benjamini–Hochberg correction was applied to the six pairwise Spearman correlations, whereas other exploratory comparisons were not adjusted for multiplicity. Retrospective protocol heterogeneity may have influenced vessel conspicuity, particularly for the LBCV, and the original clinical indications were not consistently recoverable. Sparse categories and the sample size of 100 limit inference for rare configurations. Larger multicentre studies with standardised acquisition and both categorical and metric measurements are required to establish population reference distributions and external reproducibility.

## 5. Conclusions

Within this cohort, the BCT differed markedly from the AA and LBCV: it reached or crossed the SN in 64% of cases and was fully cervical/high-riding in 49%, more often in women, whereas the AA and LBCV were cervical/high-riding in only 3% and 5% of cases, respectively. Complete manubrial joining of the SCJs was absent, so an interclavicular suprasternal space of variable degree was the rule rather than the exception. As a single-centre retrospective sample with heterogeneous CTA protocols, these are cohort frequencies rather than population-based prevalence estimates. The proposed SN-referenced ordinal classification offers a reproducible (weighted Cohen kappa = 0.89), descriptive framework for this topography; it has not been externally validated and was not designed to measure procedural outcomes. A marked interclavicular space or a high-riding BCT on preoperative CTA may be a useful anatomical cue supporting deliberate assessment of the great vessels before front-of-neck airway access, tracheostomy, or anterior cervical, thyroid, or parathyroid surgery—a potential relevance that warrants prospective evaluation.

## Figures and Tables

**Table 1:** Aortic arch (AA) position relative to the suprasternal notch (SN).

AA Category	Code	n	%	Male (n)	Female (n)	Operational Definition
Mediastinal	1	85	85.0	45	40	Aortic arch entirely below the SN reference plane
Cervicomediastinal	2	12	12.0	2	10	Aortic arch crosses the SN reference plane, partly above and partly below it
Cervical/high-riding	3	3	3.0	1	2	Aortic arch entirely above the SN reference plane
Total		100	100.0	48	52	

**Table 2:** Brachiocephalic trunk (BCT) position relative to the suprasternal notch (SN).

BCT Category	Code	n	%	Male (n)	Female (n)
Mediastinal	1	36	36.0	25	11
Cervicomediastinal	2	15	15.0	7	8
Cervical/high-riding	3	49	49.0	16	33
Total		100	100.0	48	52

**Table 3:** AA × BCT combination types.

Spreadsheet Code/Type	Operational Definition	n	%	Tier
A/Ia	Mediastinal AA + Mediastinal BCT	36	36.0	I
B/IIa	Mediastinal AA + Cervicomediastinal BCT	15	15.0	II
C/IIIa	Mediastinal AA + Cervical-HR BCT	34	34.0	III
F/IIIb	Cervicomediastinal AA + Cervical-HR BCT	12	12.0	III
I/IIIc	Cervical-HR AA + Cervical-HR BCT	3	3.0	III
Total		100	100.0	

**Table 4:** Left brachiocephalic vein (LBCV) position relative to the suprasternal notch (SN).

LBCV Category	Code	n	%	Operational Definition
Mediastinal	1	73	73.0	Entirely below SN
Cervicomediastinal	2	22	22.0	Crosses the SN level
Cervical/high-riding	3	5	5.0	Entirely above SN
Total		100	100.0	

**Table 5:** Bilateral SCJ manubrial joining grades.

SCJ Grade	Code	Right n (%)	Left n (%)	Topographic Meaning
Complete/100%	1	0 (0.0)	0 (0.0)	Classical SN topography
≥50% to <100% manubrial joining	2	36 (36.0)	35 (35.0)	Mild interclavicular component
≥25% to <50% manubrial joining	3	49 (49.0)	52 (52.0)	Clear interclavicular component
<25% manubrial joining	4	15 (15.0)	13 (13.0)	Marked suprasternal–interclavicular space
Total		100 (100)	100 (100)	

**Table 6:** Topographic interpretation of the suprasternal region.

Topographic Interpretation	n	%
Mild suprasternal–interclavicular component	33	33.0
Clear suprasternal–interclavicular component	52	52.0
Marked suprasternal–interclavicular space	15	15.0
Total	100	100.0

## Data Availability

The datasets used and analysed during the current study are available from the corresponding author upon reasonable request.

## Supplementary Materials

The following supporting information can be downloaded at: https://www.mdpi.com/article/10.3390/diagnostics16172724/s1. Figure S1: SN-referenced ordinal grading, combined tier rule, and AA × BCT codebook; Figure S2: schematic of all 26 observed four-variable topographic types; Table S1: global four-variable topographic types; Table S2: clinical and epidemiological features of classical cervical aortic arch; Table S3: bovine aortic arch pooled prevalence and outcome data.

## Abbreviations

The following abbreviations are used in this manuscript:

AA	aortic arch
BCT	brachiocephalic trunk
CAA	cervical aortic arch
CI	confidence interval
CT	computed tomography
CTA	computed tomography angiography
CVC	central venous catheterisation
df	degrees of freedom
DICOM	Digital Imaging and Communications in Medicine
HR	high-riding
LBCV	left brachiocephalic vein
MDCT	multi-detector computed tomography
MPR	multiplanar reformation
OR	odds ratio
SCJ	sternoclavicular joint
SD	standard deviation
SN	suprasternal notch
TAVR	transcatheter aortic valve replacement
