# Supplementary material for: Major Vessels of the Interclavicular Suprasternal Space: A Suprasternal Notch-Referenced Topographic Classification with Potential Procedural Relevance
Source: Diagnostics (Basel). 2026 Aug 26;16(17):2724. doi: 10.3390/diagnostics16172724 (PMC13565290; doi:10.3390/diagnostics16172724)
Supplement: Supplementary file 1 [file diagnostics-16-02724-s001.zip › diagnostics-4454576_Figure_S1_v03.pdf]

## Supplementary Figure S1

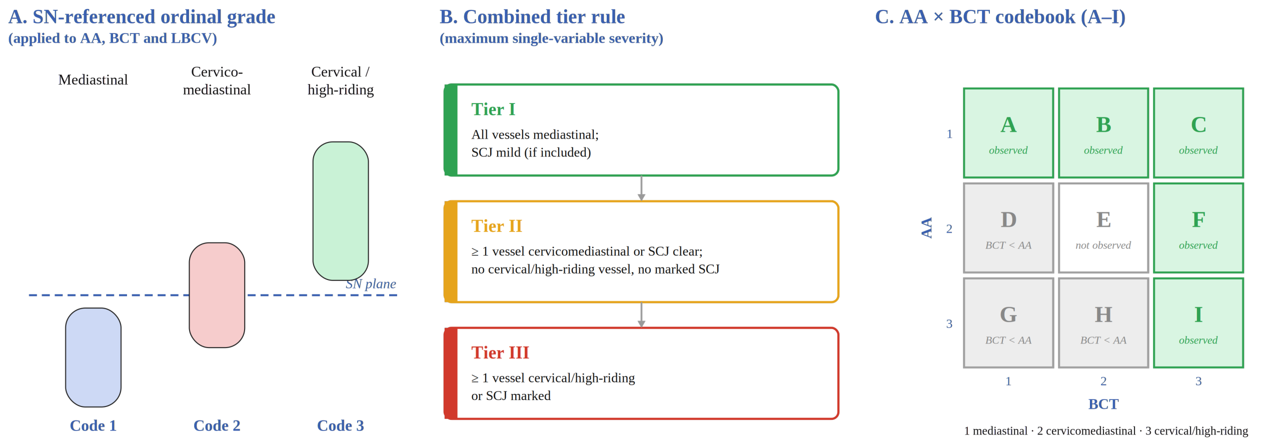


Figure S1. SN-referenced ordinal grading, combined tier rule, and AA × BCT codebook. (A) Each vessel (AA, BCT, LBCV) is graded relative to the horizontal SN plane as code 1 (mediastinal), code 2 (cervicomediastinal) or code 3 (cervical/high-riding). (B) Each pattern is assigned to a tier by its most severe single variable. (C) The nine AA × BCT combinations are labelled A–I; those observed (A, B, C, F, I) are green, D/G/H (BCT lower than AA) are anatomically implausible (grey), and E was not observed (white). Tier assignment reflects maximum topographic grade only and does not represent measured procedural risk.
