# Supplementary material for: Major Vessels of the Interclavicular Suprasternal Space: A Suprasternal Notch-Referenced Topographic Classification with Potential Procedural Relevance
Source: Diagnostics (Basel). 2026 Aug 26;16(17):2724. doi: 10.3390/diagnostics16172724 (PMC13565290; doi:10.3390/diagnostics16172724)
Supplement: Supplementary file 1 [file diagnostics-16-02724-s001.zip › diagnostics-4454576_Figure_S2_v03.pdf]

Supplementary Figure S2

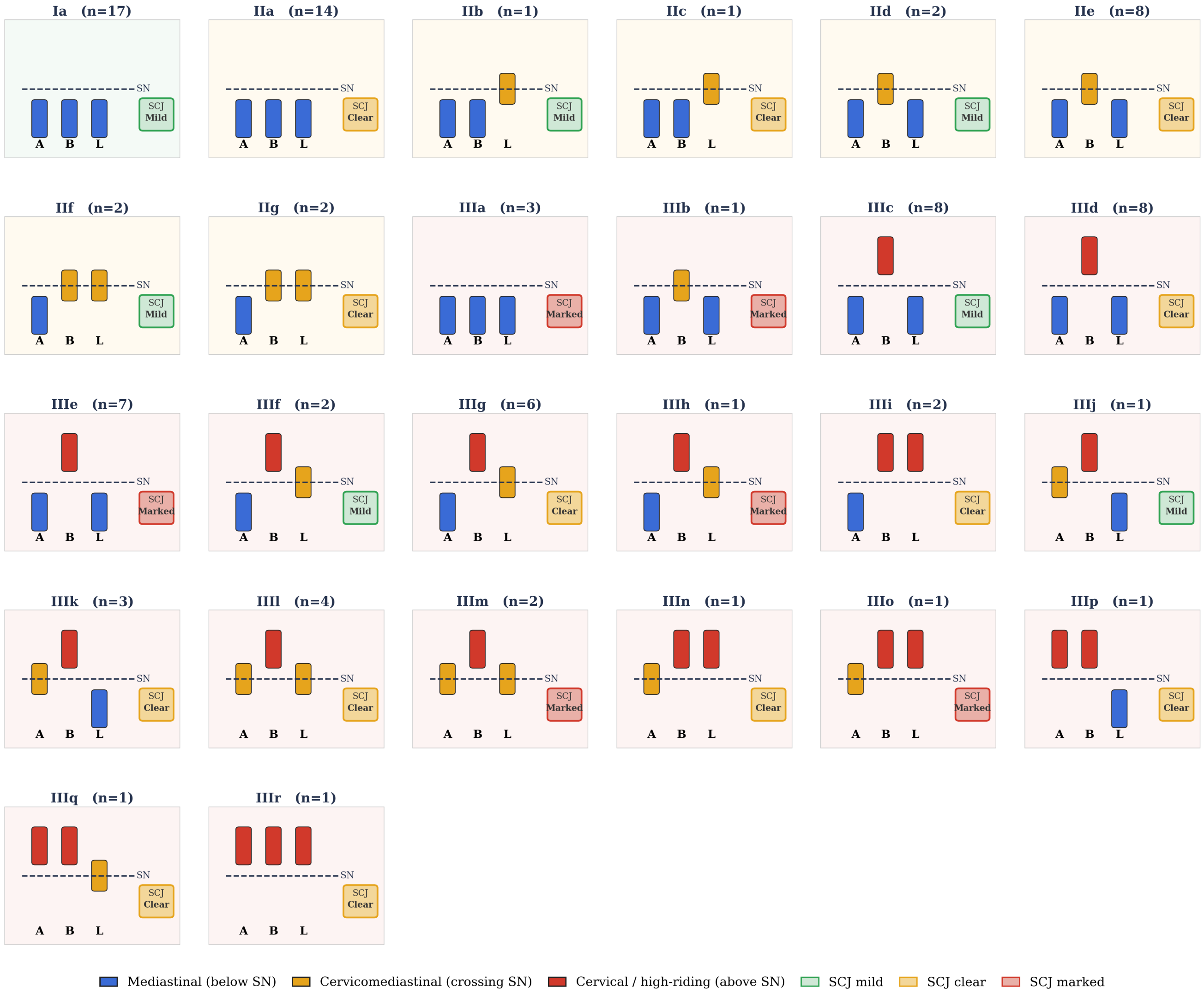

Figure S2. Schematic of all 26 observed four-variable topographic types (Table S1). Each panel shows AA (A), BCT (B) and LBCV (L) relative to the SN plane with the integrated SCJ grade; bars are coloured by level and the panel tint denotes tier. n = cases. The panels are descriptive summaries only; individual types were not subjected to inferential testing.
