# Supplementary material for: Major Vessels of the Interclavicular Suprasternal Space: A Suprasternal Notch-Referenced Topographic Classification with Potential Procedural Relevance
Source: Diagnostics (Basel). 2026 Aug 26;16(17):2724. doi: 10.3390/diagnostics16172724 (PMC13565290; doi:10.3390/diagnostics16172724)
Supplement: Supplementary file 1 [file diagnostics-16-02724-s001.zip › diagnostics-4454576_Table_S1_v03.pdf]

Supplementary Materials

Major Vessels of the Interclavicular Suprasternal Space: A Suprasternal Notch-Referenced Topographic Classification with Potential Procedural Relevance

Ionuț Mădălin Munteanu, Adelina Maria Jianu, Răzvan Costin Tudose, Mugurel Constantin Rusu, Alexandra Diana Vrapciu, George Triantafyllou, Maria Piagkou and Iulian Brezean

Table S1. Global four-variable topographic types of the suprasternal-interclavicular region (100 CTA cases). Vascular variables are classified relative to the suprasternal notch (SN) reference plane; SCJ topography is the integrated bilateral grade. Tier I, all vascular variables mediastinal with mild SCJ; Tier II, cervicomediastinal vascular or clear SCJ without any cervical/high-riding vessel or marked SCJ; Tier III, at least one cervical/high-riding vessel or marked SCJ. AA, aortic arch; BCT, brachiocephalic trunk; LBCV, left brachiocephalic vein; SCJ, sternoclavicular joint; HR, high-riding. The 26 types are descriptive only; no inferential testing was applied to individual four-variable types.

Type	AA	BCT	LBCV	SCJ	n
Ia	Mediastinal	Mediastinal	Mediastinal	Mild	17
IIa	Mediastinal	Mediastinal	Mediastinal	Clear	14
IIb	Mediastinal	Mediastinal	Cervicomediastinal	Mild	1
IIc	Mediastinal	Mediastinal	Cervicomediastinal	Clear	1
IId	Mediastinal	Cervicomediastinal	Mediastinal	Mild	2
IIe	Mediastinal	Cervicomediastinal	Mediastinal	Clear	8
IIf	Mediastinal	Cervicomediastinal	Cervicomediastinal	Mild	2
IIg	Mediastinal	Cervicomediastinal	Cervicomediastinal	Clear	2
IIIa	Mediastinal	Mediastinal	Mediastinal	Marked	3
IIIb	Mediastinal	Cervicomediastinal	Mediastinal	Marked	1
IIIc	Mediastinal	Cervical-HR	Mediastinal	Mild	8
IIId	Mediastinal	Cervical-HR	Mediastinal	Clear	8
IIIe	Mediastinal	Cervical-HR	Mediastinal	Marked	7
IIIf	Mediastinal	Cervical-HR	Cervicomediastinal	Mild	2
IIIg	Mediastinal	Cervical-HR	Cervicomediastinal	Clear	6
IIIh	Mediastinal	Cervical-HR	Cervicomediastinal	Marked	1
IIIi	Mediastinal	Cervical-HR	Cervical-HR	Clear	2
IIIj	Cervicomediastinal	Cervical-HR	Mediastinal	Mild	1
IIIk	Cervicomediastinal	Cervical-HR	Mediastinal	Clear	3
IIIl	Cervicomediastinal	Cervical-HR	Cervicomediastinal	Clear	4
IIIm	Cervicomediastinal	Cervical-HR	Cervicomediastinal	Marked	2
IIIn	Cervicomediastinal	Cervical-HR	Cervical-HR	Clear	1
IIIo	Cervicomediastinal	Cervical-HR	Cervical-HR	Marked	1
IIIp	Cervical-HR	Cervical-HR	Mediastinal	Clear	1
IIIq	Cervical-HR	Cervical-HR	Cervicomediastinal	Clear	1
IIIr	Cervical-HR	Cervical-HR	Cervical-HR	Clear	1
