# Supplementary material for: Major Vessels of the Interclavicular Suprasternal Space: A Suprasternal Notch-Referenced Topographic Classification with Potential Procedural Relevance
Source: Diagnostics (Basel). 2026 Aug 26;16(17):2724. doi: 10.3390/diagnostics16172724 (PMC13565290; doi:10.3390/diagnostics16172724)
Supplement: Supplementary file 1 [file diagnostics-16-02724-s001.zip › diagnostics-4454576_Table_S2_v03.pdf]

Supplementary Materials

Major Vessels of the Interclavicular Suprasternal Space: A Suprasternal Notch-Referenced Topographic Classification with Potential Procedural Relevance

Ionuț Mădălin Munteanu, Adelina Maria Jianu, Răzvan Costin Tudose, Mugurel Constantin Rusu, Alexandra Diana Vrapciu, George Triantafyllou, Maria Piagkou and Iulian Brezean

Table S2. Clinical and epidemiological features of classical cervical aortic arch (CAA), based on published data. Full references are given in the main manuscript.

Feature	Data	Reference
Arch laterality	Left-sided in 85.7% of surgical cases	Zhong et al. (2020)
Sex predominance	65–67% female	Zhong et al. (2020); Baudo et al. (2024)
Mean age	34–38 years	Zhong et al. (2020); Baudo et al. (2024)
Asymptomatic at diagnosis	45–52%	Zhong et al. (2020); Baudo et al. (2024)
Associated aneurysm	62% (adult case review)	Baudo et al. (2024)
Operative mortality	0% (35-patient series)	Zhong et al. (2020)
6-year event-free survival	93.5%	Zhong et al. (2020)
Classification system	Haughton 5-type A–E (1975)	Haughton et al. (1975)
