# Supplementary material for: Major Vessels of the Interclavicular Suprasternal Space: A Suprasternal Notch-Referenced Topographic Classification with Potential Procedural Relevance
Source: Diagnostics (Basel). 2026 Aug 26;16(17):2724. doi: 10.3390/diagnostics16172724 (PMC13565290; doi:10.3390/diagnostics16172724)
Supplement: Supplementary file 1 [file diagnostics-16-02724-s001.zip › diagnostics-4454576_Table_S3_v03.pdf]

Supplementary Materials

Major Vessels of the Interclavicular Suprasternal Space: A Suprasternal Notch-Referenced Topographic Classification with Potential Procedural Relevance

Ionuț Mădălin Munteanu, Adelina Maria Jianu, Răzvan Costin Tudose, Mugurel Constantin Rusu, Alexandra Diana Vrapciu, George Triantafyllou, Maria Piagkou and Iulian Brezean

Table S3. Bovine aortic arch: pooled prevalence and selected clinical outcome data. TAVR, transcatheter aortic valve replacement; OR, odds ratio; HR, hazard ratio; CI, confidence interval. Full references are given in the main manuscript.

**Parameter**	**Finding**	**Reference**
Pooled adult prevalence (meta-analysis, n = 26,721)	13.6% overall	Popieluszko et al. (2018)
Prevalence in African-ancestry populations	Up to 26.8%	Popieluszko et al. (2018)
Subtype: common BCT–left common carotid origin	~13% of adults	Murray & Meguid (2023)
Subtype: left common carotid arising from BCT	~9% of adults	Murray & Meguid (2023)
Arch anomalies in thoracic aortic disease vs controls	33.5% vs 18.2% ($p < 0.001$)	Dumfarth et al. (2015)
TAVR periprocedural stroke	Adjusted OR 2.16 (95% CI 1.22–3.83)	Lo Russo et al. (2024)
TAVR long-term stroke	Adjusted HR 2.11 (95% CI 1.51–2.93)	Lo Russo et al. (2024)
